# Electrospraying of Bio-Based Chitosan Microcapsules Using Novel Mixed Cross-Linker: Experimental and Response Surface Methodology Optimization

**DOI:** 10.3390/ma15238447

**Published:** 2022-11-27

**Authors:** Lydia Uko, Hussien Noby, Abdelrahman Zkria, Marwa ElKady

**Affiliations:** 1 Chemical and Petrochemicals Engineering, Egypt-Japan University of Science and Technology, Alexandria 21934, Egypt; 2 Materials Engineering and Design, Faculty of Energy Engineering, Aswan University, Aswan 81528, Egypt; 3 Department of Applied Science for Electronics and Materials, Kyushu University, Kasuga, Fukuoka 816-8580, Japan; 4 Department of Physics, Faculty of Science, Aswan University, Aswan 81528, Egypt; 5 Fabrication Technology Department, Advanced Technology and New Materials Research Institute (ATNMRI), City of Scientific Research and Technology Applications, Alexandria 21934, Egypt

**Keywords:** chitosan, electrospraying, microcapsules, surface responses methodology, optimization

## Abstract

Chitosan microcapsules draw attention due to their biodegradability, biocompatibility, antibacterial behavior, low cost, easy processing, and the capability to be used for different applications. This study utilized the electrospraying technique for the chitosan microcapsules formulation. As a novel cross-linking agent, a mixture of oxalic acid and sodium phosphate dibasic was utilized as a collecting solution for the first time in the electrospraying of chitosan microcapsules. Scanning Electron Microscopy (SEM) was utilized to optimize the spherical morphology and size of the experimentally obtained microcapsules. The different parameters, including chitosan concentration, applied voltage, flow rate, and tip-to-collector (TTC) distance, affecting the microcapsules’ size, sphericity, yield, and combined effects were optimized using Surface Responses Methodology (RSM). The Analysis of Variance (ANOVA) was utilized to obtain the impact of each parameter on the process responses. Accordingly, the results illustrated the significant impact of the voltage parameter, with the highest F-values and least *p*-values, on the capsule size, sphericity, and yield. The predicted optimum conditions were determined as 5 wt% chitosan concentration, 7 mL/h flow rate, 22 kV, and 8 cm TTC distance. The predicted responses at the optimized conditions are 389 µm, 0.72, and 80.6% for the capsule size, sphericity, and yield, respectively. While the validation of the model prediction was conducted experimentally, the obtained results were 369.2 ± 23.5 µm, 0.75 ± 0.04, and 87.3 ± 11.4%, respectively. The optimization process was successfully examined for the chitosan microcapsules manufacturing.

## 1. Introduction

Particle size and morphology are essential in several industrial and scientific processes, especially micro-/nanotechnology, and dictate the developed product’s quality, behavior, durability, and effectiveness. Controlling these crucial requirements is challenging in conventional microcapsules fabrication techniques. The electrospraying technique is a critical approach to overcoming this drawback [1]. Electrospraying, also known as electrohydrodynamic spraying, is a liquid atomization method using applied electrical forces [2,3]. When a liquid droplet leaves a capillary nozzle connected to an electric field via a high voltage, its interface is deformed. Then, an electrostatic force is generated inside the droplet through the electric charge that competes with the droplet’s surface tension, forming a stable cone-jet mode called Taylor cone, usually a charged droplet. Eventually, the electrostatic force overcomes the droplet’s surface tension, dispersing it into fine droplets. Because of the Coulomb repulsion of the charges, the droplets do not coalesce as they travel toward the collector [1,4]. Electrospraying is employed in producing micro- or nanoparticles, micro- or nanocapsules, and deposition of micro- and nano-thin-film. In the fabrication of microcapsules, the electrospraying technique is gaining greater recognition because of its superior advantages: simple operation, cost-effectiveness, little or no solvents, no need for surfactant, and low-temperature process [4]. Moreover, electrospraying has a combinable conductive capillary nozzle, adjustable parameters, and replaceable collectors [5,6].

Several natural biobased polymers, such as pectin, sodium alginate, cellulose, and chitosan, have been processed into micro-/nanocapsules using the electrospraying technique. The prepared capsules were utilized for drug delivery [5,7], textile production [8], food [9] and innovative coatings for anticorrosion [10,11]. Chitosan microcapsules were also utilized for the abovementioned applications because of their desirable properties: biocompatibility, biodegradability, antibacterial activity, low cost, and easy film formation [12]. Chitosan is a linear polysaccharide consisting mainly of D-glucosamine and N-acetyl glucosamine units obtained from the deacetylation of chitin [13,14]. It requires covalent or ionic cross-linking, which bonds the chains when reacting with specific reactive sites in its structural units and the cross-linking agent to form stable microcapsules [8]. Coacervation/precipitation, ionic gelation, emulsion droplet coalescence, emulsion cross-linking reaction, and spray-drying processes were tested for chitosan microcapsules fabrication [15]. Selecting any method depends on factors like particle size requirement, chemical and thermal stability, and the toxicity level associated with the final product [16].

In the chitosan microcapsules electrospraying, ionic cross-linking is majorly employed to allow strong electrostatic attraction between the cationic regions of chitosan (protonated sites—${\mathrm{NH}}_{3}^{+}$) and the cross-linker’s anionic regions [17]. Ionic cross-linking is simple and practical because it requires only contacting chitosan with the cross-linking agent. Tripolyphosphate has been predominantly used as chitosan’s anionic cross-linking agent because of its multivalency, as reported in some of the literature [18,19]. Exploration of other cross-linkers which may be beneficial to the cross-linking of chitosan for microcapsule production through the electrospraying technique is an open field of innovation. The electrospraying process can produce chitosan capsules size in the micro and nano ranges. The desirable size, low polydispersity, or mono-dispersity can be achieved when the proper parameters are chosen. This is possible at reduced flow rates, moderate conductivity of the electrosprayed polymer solution, increased polymer concentration, and medium applied voltage [4,5]. Obtaining the right capsule size by tailoring the process parameters is always a complex venture, possible only through establishing the stable-cone jet mode, which highly depends on voltage and flow rate [20].

In the several reported cases where chitosan microcapsules were produced, the size, shape, or yield of capsules are appraised as essential parameters for the application. Obtaining the appropriate size, shape, or optimum yield gives credibility to production desirability. Particle size is a vital consideration for applications such as ceramic coatings, paints, and powder in cosmetic and pharmaceutical industries and self-healing applications for anticorrosion protection [2]. For instance, the self-healing performance in the anticorrosion application is influenced by the size and content of the capsule. By establishing a relationship between microcapsule size and microcapsule weight fractions, it was possible to develop self-healing systems that repaired specific types of corrosion damage [21]. Size affects the toughness of microcapsules and the interface between them and the coating matrix in which they are incorporated. It was reported that smaller-sized capsules showed greater toughness than larger ones [22], but the capsules with larger sizes provided better healing performance against corrosion [23]. In biomedical applications, size influences properties such as bioavailability, cellular uptake, and blood circular time [24]. In addition, Chen et al. reported using millimeter-sized capsules to reduce the thromboembolic risk in the control of hemorrhage [25].

Besides, the sphericity of the microcapsules is a crucial consideration. The polymer solution parameters, such as solubility, concentration, molecular weight, solvent miscibility, and solution conductivity, play a vital role in sphericity. Polymer concentration and molecular weight are linked to the shape of the capsule, where decreased concentration or increased molecular weight leads to non-spherical shapes such as oblong, wrinkled, tear-shaped, or tailed capsules. To obtain spherical capsules, sufficient polymer entanglements must be present [4]. Sphericity was a crucial requirement in self-healing applications where sphericity improved properties such as mechanical stability and strength of the microcapsules [21,26]. Spherical microcapsules also provided smoother surfaces [21,25]. Generally, the size, sphericity, and yield of microcapsules generated by electrospraying may be influenced by process parameters such as voltage, needle size, flow rate, and TTC distance, along with solution parameters. Hence, desirable microcapsule size, sphericity, and yield might be obtained by optimizing all necessary parameters. For instance, alginate micro-/macro-capsules were processed by electrospraying to study the parameters affecting the size and sphericity or shape of the capsules [27,28,29]. Others fabricated chitosan nano-/microcapsules reported studying the size and size distribution [18,24,30]. In addition, the yield of chitosan microcapsules produced by the electrospraying technique was investigated by a few studies [15,18]. To the best of the authors’ knowledge, no one has yet reported the parameters optimization that simultaneously affect the size, yield, and sphericity of chitosan microcapsules formulated by the electrospraying technique. Moreover, this investigation deals with the utilization of the novel mixed cross-linker agent of oxalic acid and sodium phosphate dibasic salt for the first time for chitosan capsule formulation using the electrospraying technique. The new cross-linker provided the anionic regions that interacted with the cationic regions of chitosan to form ionically cross-linked chitosan microcapsules. The carboxyl and phosphate groups of the cross-linker produced multivalent ions, which facilitated the instant formation of microcapsules on the introduction of chitosan droplets into the cross-linking solution.

Accordingly, this study examined the effect of chitosan concentration, flow rate, TTC distance, and the applied voltage on the size, sphericity, and yield of chitosan microcapsules produced by the electrospraying technique. The electrosprayed capsules were collected with a novel cross-linking solution. Response surface methodology (RSM) was utilized as a befitting mathematical and statistical tool to achieve process parameters optimization.

## 2. Materials and Methods

### 2.1. Materials

Chitosan with molecular weight 100,000 Da–300,000 Da (ACROS Organics, USA), glacial acetic acid (≥99% in volume) purchased from Sigma-Aldrich (Germany), oxalic acid (Fluka Analytical), and sodium phosphate dibasic heptahydrate (Fisher Bioreagents, India) were the materials utilized. The chemicals were of analytical grade and used without additional purification.

### 2.2. Preparation of Chitosan Microcapsules by Electrospraying Technique

Chitosan solutions of different concentrations were prepared by adding chitosan powder in amounts of 4, 4.5, 5, 5.5, and 6 g in 0.8, 1.2, 1.5, 1.7, and 2 *v*/*v*% acetic acid concentrations to obtain chitosan solutions in *w*/*v*%. Dissolution took place under magnetic stirring at room temperature until homogenized chitosan solutions were obtained. The solutions were left stationary overnight for complete degassing. In addition, the cross-linking solution was developed by processing a mixture of oxalic acid and sodium phosphate dibasic solids into a colorless solution. Then, the chitosan solutions were drawn into a 10 mL syringe attached with a blunt-tipped metal needle of 25 G held constant and subsequently hooked to the digitally controlled syringe pump of the electrospraying machine, which has a variable voltage of 0–60 kV (NanoNC, Korea). The solution was electrosprayed at applied voltages varied in the range of 10–30 kV and at varying flow rates into the cross-linking solution in a beaker at various distances of 7–11 cm from the needle tip. The ratio of the volume of chitosan solution sprayed to that of the cross-linker was fixed at 0.3 for all the experiments. The cross-linking solution was placed on a magnetic stirrer rotating at a speed of 120 rpm. The formed microcapsules were left to cross-link for 12 h before washing with distilled water and air-dried.

### 2.3. Experimental Design for Electrospraying

To determine the best parameters for better microcapsules’ size, sphericity, and higher yield, optimization was conducted using the Response Surface Methodology and central composite design (RSM-CCD). The range of parameters for the design was chosen based on the preliminary experiments. Chitosan concentration, flow rate, voltage, and tip-to-collector (TTC) distance were the relevant independent variables affecting responses such as microcapsule size, sphericity, and yield. A central composite design (Five levels) and quadratic/2FI model were utilized to design the experiments. The RSM-CCD design displaying the coded and uncoded levels and the range used in the experiments are shown in Table 1. Thirty runs, including sixteen fractional factorial points, six central points, and eight axial points, were randomly performed to optimize the process parameters. The experiments were carried out according to the actual experimental design matrix. The actual levels of the factors were coded using Equation (1):

(1) $$Z=\frac{Z_{O}-Z_{C}}{\Delta Z},$$

where Z and Z_o_ represent coded and real levels of the factors, respectively. ∆Z shows the step change, while Z_c_ represents the actual value at the central point. The equations for each studied factor were developed from the above equation to code their actual levels. Design Expert software (version 13.0.12, StatEase^®^) was used to establish the design.

Multiple factorial regression was used to show the predicted responses in each trial as a function of the independent variables as given by the equations Equation (2) for microcapsule size and sphericity and Equation (3) for yield:

(2) $$Y_{\left(Quadratic\right)}=\beta_{o}+\beta_{1}Y_{1}+\beta_{2}Y_{2}+\beta_{3}Y_{3}+\beta_{4}Y_{4}+\beta_{12}Y_{1}Y_{2}+\beta_{13}Y_{1}Y_{3}+\beta_{14}Y_{1}Y_{4}+\beta_{23}Y_{2}Y_{3}+\beta_{24}Y_{2}Y_{4}-\beta_{34}Y_{3}Y_{4}+\beta_{11}Y_{1}^{2}+\beta_{22}Y_{2}^{2}+\beta_{33}Y_{3}^{2}+\beta_{44}Y_{4}^{2}$$

*Y_(2FI)_* = β_o_ + β_1_Y_1_ + β_2_Y_2_ + β_3_Y_3_ + β_4_Y_4_ + β_12_Y_1_Y_2_ + β_13_Y_1_Y_3_ + β_14_Y_1_Y_4_ + β_23_Y_2_Y_3_ + β_24_Y_2_Y_4_ − β_34_Y_3_Y_4_ (3)

where Y represents the response variables, β_i_, β_ii_, and β_ij_ are the estimated linear, quadratic, and interaction coefficients of the equation, respectively. β_0_ is the interception coefficient, while Y_1_, Y_2_, Y_3_, and Y_4_ are the manipulated parameters, i.e., concentration, flow rate, voltage, and TTC distance, Y_1_Y_2_, Y_1_Y_3_, Y_1_Y_3_, etc., are the interaction terms, and $Y_{1}^{2}$, $Y_{2}^{2}$, etc. are the polynomial terms.

### 2.4. Morphology of Microcapsules

The air-dried chitosan microcapsules were characterized using a scanning electron microscope (SEM) (JCM-6000Plus; JEOL Electronics Co. Ltd., Tokyo, Japan) after placing the capsules on the adhesive tape with an accelerating voltage of 15 kV.

### 2.5. Size Analysis of Electrosprayed Microcapsules

The obtained SEM of the microcapsules was used for the microcapsule size analysis, determined using the FIJI software (Java 8 bundled). The mean microcapsule size was obtained by analyzing about 25 microcapsules from each sample run by taking each microcapsule’s major, minor, and orthogonal lengths (diameters).

### 2.6. Determination of Microcapsules’ Sphericity

Some studies [28,29,31] have reported using aspect ratio (AR) (d_max_/d_min_), sphericity factor (d_max_ − d_min_/d_max_ + d_min_), and sphericity coefficient (d_min_/d_max_) to estimate the sphericity of alginate microcapsules. The sphericity of the chitosan microcapsules was determined using Equation (4):

(4) $$\mathrm{Sphericity}=\frac{D_{minor}}{D_{major}}$$

where *D_minor_* is the mean value of the shortest distance (diameter), and D*_major_* is the mean value of the longest distance (diameter) of the microcapsule estimated from the 2D image of the microcapsules [27]. The sphericity was estimated from approximately 25 random samples for each experimental run. The sphericity is determined between 0 and 1, and a value close to 1 is considered spherical.

### 2.7. Microcapsule Yield Analysis

In the electrospraying technique, process and solution parameters significantly affect the yield. Ardila et al. [15] estimated yield as the number of particles per collector surface unit area, where a stainless plate was utilized as a collector for the chitosan microcapsules. In a situation where the collector is a cross-linking solution, this method may not be sufficient. Several pieces of literature have calculated yield in different forms for different microencapsulation techniques. In the spray drying process, the yield was estimated as the mass of product powders recovered from the equipment at the end of the process to the mass of the solid content of the initial solution fed into the spray dryer chamber [32].

While processing chitosan microcapsules by electrospraying, Xu & Hanna [18] estimated the yield as the mass of dried loaded particles to the total initial mass of polymer and encapsulated material. This method was used in this study to estimate yield with some modifications as shown in Equation (5):

(5) $$\mathrm{Yield}=(\frac{m_{dc}}{m_{ti}})\times 100$$

where m*_dc_* is the mass of dried capsules; m*_ti_*(mass of total ingredients) = m*_i_* + m_c_; m*_i_* (mass of ingredients) = conc. × vol. (C × v) and m*_c_* (mass of cross-linker) = m*_dc_ –* m*_i_*.

### 2.8. Statistical Analysis

ANOVA was employed to validate the statistical fitness of the polynomial equations generated. The responses predicted were fitted concurrently to different models. The best fitting experimental model, such as linear, quadratic, and interaction, was chosen statistically based on a comparison of various statistical parameters like R^2^ (multiple correlation coefficient), predicted R^2^ (Pred. R^2^), CV (coefficient of variation) and adjusted R^2^ (Adj. R^2^). The *p*-value < 0.05 established the level of significance.

### 2.9. Validation of Model and Optimization

Model confirmation analysis was conducted for the predicted optimized responses (microcapsule size, sphericity, and yield) conditions. An equal importance level of 3 out of 5 was assigned to all the parameters with the desirable outcome of minimizing microcapsule size, maximizing sphericity, and maximizing yield.

## 3. Results

### 3.1. Statical Modeling/Experimental Design

RSM-CCD analysis was used to guide the production of chitosan microcapsules and to demonstrate the relationship between microcapsule size, sphericity, and yield with the chosen parameters, i.e., chitosan solution concentration, flow rate, voltage, and TTC distance. The effect of the variables on the studied responses was investigated. The calculated results of the responses as a function of the independent variables are displayed in Table 2.

**Figure 1:**
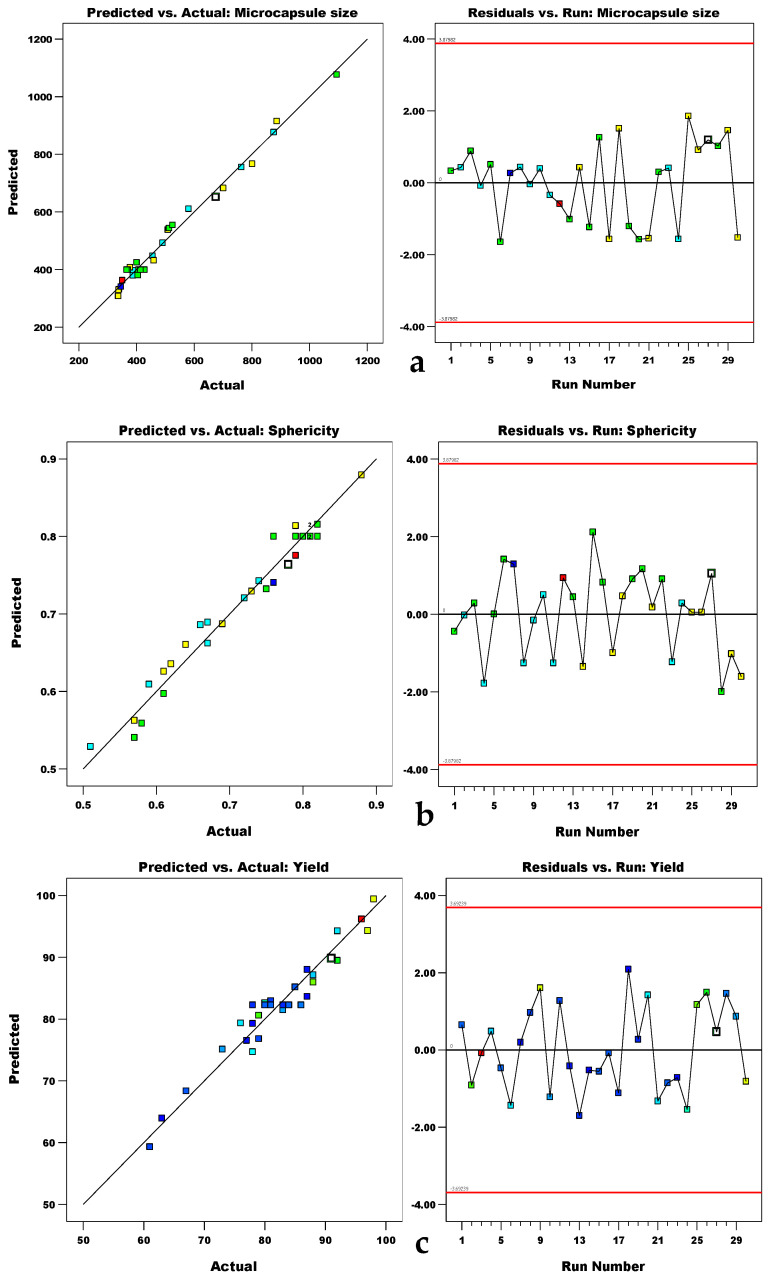
Linear correlation plots for predicted and experimental values and residual plots for (**a**) microcapsule size, (**b**) sphericity, and (**c**) yield.

The responses studied in this work (microcapsule size, sphericity, and yield) were fitted to the models, which were statistically analyzed and deemed to be significant (*p*-values < 0.001). Hence, the models could be utilized to navigate the design space. The models’ quality was judged by comparing the predicted and experimental values. Because the lack-of-fit was acceptable for all the responses, the models were appropriate for predicting and optimizing the electrospraying process, as shown in Figure 1. The spread of dependent variables under the experimental conditions is confirmed in the scatter plot of the residuals.

The values of the response variables were predicted from coefficients of polynomial equation computed from experimental data. The quadratic regression equations for microcapsule size and sphericity, and 2FI for yield are stated in Equations (6)–(8):

(6) $$Microcapsule\;size(\;mm)=7461.68\;-\;1529.63Y_{1}\;-\;54.4689Y_{2}\;-\;328.725Y_{3}+210.343Y_{4}+0.269231Y_{1}Y_{2}+33.15Y_{1}Y_{3}\;+17Y_{1}Y_{4}+1.3Y_{2}Y_{3}\;-\;1.01923Y_{2}Y_{4}\;-\;3.075Y_{3}Y_{4}+62.8333Y_{1}^{2}+1.20611Y_{2}^{2}+3.51333Y_{3}^{2}\;-\;11.9167Y_{4}^{2}$$

(7) $$Sphericity=-6.93436+2.37038Y_{1}+0.0420069Y_{2}+0.0292564Y_{3}+0.286827Y_{4}\;-\;0.00826923Y_{1}Y_{2}+0.00075Y_{1}Y_{3}\;\;-\;0.00375Y_{1}Y_{4}+0.00140385Y_{2}Y_{3}\;-\;0.00240385Y_{2}Y_{4}\;-\;0.001875Y_{3}Y_{4}-0.222083Y_{1}^{2}\;-\;0.000308185Y_{2}^{2}\;\;-\;0.00122083Y_{3}^{2}\;-\;0.0105208Y_{4}^{2}$$

*Yield* = 285.589 – 42.4103Y_1_ – 6.72756Y_2_ – 5.03013Y_3_ − 0.879808Y_4_ + 1.40385Y_1_Y_2_ + 0.925Y_1_Y_3_ + 0.875Y_1_Y_4_ − 0.0480769Y_2_Y_3_ + 0.00961538Y_2_Y_4_ − 0.0375Y_3_Y_4_ (8)

### 3.2. Statical Analysis (ANOVA)

The statistical analysis results are given in Table 3 and Table 4. The results show that the experimental data could be well represented with the quadratic and 2FI polynomial models, as indicated by the statistical fitness of the models in Table 4. The coefficient of determination (R^2^) is an essential factor for investigating the models that explain the proportion of the total variability by the regression model and measure the extent of response variation. It increases when a new term is added to the model, regardless of whether it is statistically significant or otherwise. If the R^2^ is closer to unity, then it means that the models are a good fit for the actual data. Otherwise, the responses are not suitable to describe the variation in the behavior [33]. The R^2^ values for microcapsule size, sphericity, and yield are 0.9875, 0.9687, and 0.9422, respectively, demonstrating that the effect of concentration, flow rate, voltage, and TTC distance on the responses can be described via quadratic and 2FI models. Adjusted R^2^ is a type of R^2^ for a certain number of terms in the model and will only increase if the newly added term improves the model and will decrease if otherwise [34].

Predicted R^2^ implies that the model predicts the response for new observations perfectly. The *p*-values determine whether a model is significant or otherwise. A *p*-value less than 0.05 shows that the factors significantly impact the responses, indicating that the response models are significant. The lack of fit at a *p*-value ≤ 0.05 was non-significant relative to the pure error for all variables showing the models are statistically accurate. A large F-value (above 4.0) indicates a highly significant influence of any term on the response. Altogether, the ANOVA parameters employed to diagnose the fitness of the independent variable to the models proved them valid.

To further measure how accurately the response models represent the experimental data, error function analysis was carried out. The equations of the error functions are given in a literature [35]. Among these error function equations, the mean absolute percentage error (MAPE) was chosen because it is considered the best-recognized practical metric for the accuracy of a model [36]. A MAPE value of less than 10% shows that the model is highly accurate, 11–20% indicates a good model, while 21–50% suggests a reasonable model [37]. The MAPE for the models is presented in Table 5. The values indicate that the models are highly accurate and are a good fit for the experimental data since the values are all below 10%.

### 3.3. Effect of the Independent Variables and Interactions on the Response Variables

Cross-linked chitosan microcapsules, with the newly developed cross-linker, were successfully prepared by electrospraying technique using different levels of the independent variables (Table 1). The regression coefficients for microcapsule size, sphericity, and yield are displayed in Table 6. The regression coefficients indicate the change expected in response to any change in the value of an independent variable (factor) when all the other factors are kept constant. The stars show the significance level for each of the coefficients, where the three stars, two stars, and one star represent significance at *p*-values less than 0.001 (***), 0.01 (**), and 0.05 (*), respectively. The negative sign beside the coefficient suggests that an increase in the independent variable leads to a decrease in the response variable and vice versa [38].

#### 3.3.1. Microcapsule Size

**Figure 2:**
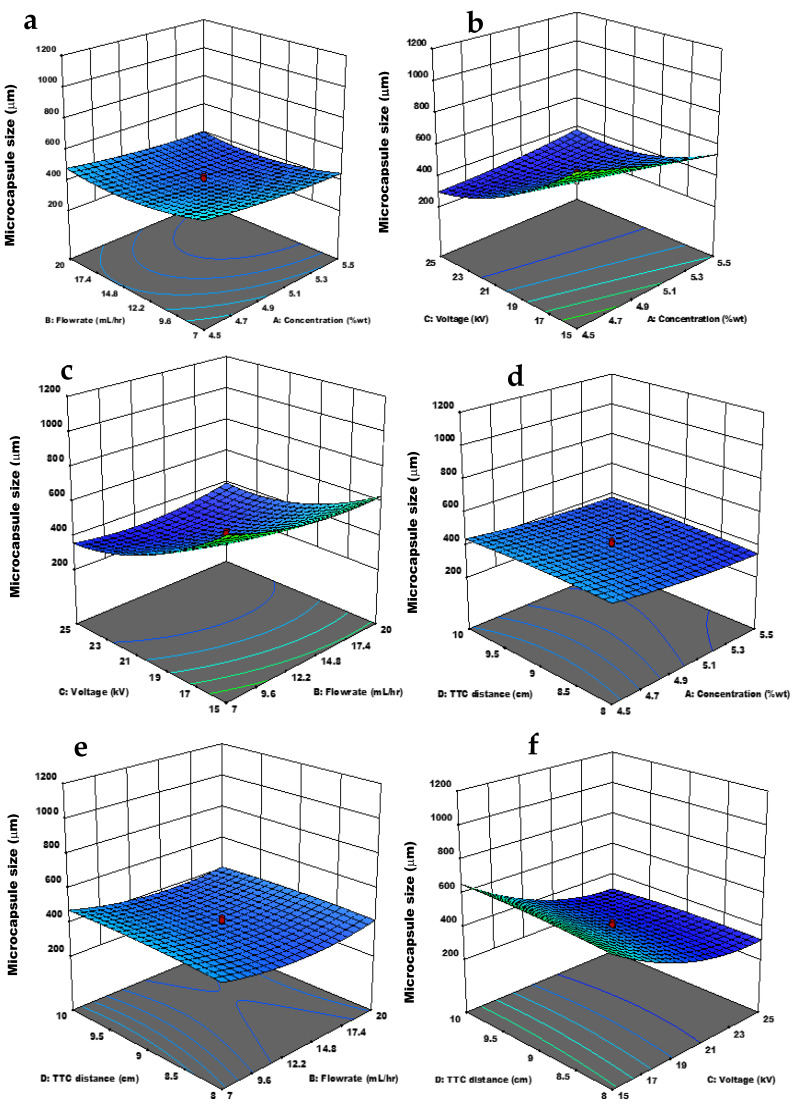
Response surface plot of the interaction effect of independent variables on microcapsule size: (**a**) Flow rate and concentration; (**b**) Voltage and concentration, (**c**) Voltage and flow rate; (**d**) TTC distance and concentration; (**e**) TTC distance and flow rate; (**f**) TTC distance and voltage.

The effect of the independent and interactive variables on microcapsule size is depicted in Figure 2. Higher polymer concentration forms viscous emulsion and, consequently, larger microcapsules [39]. In this study, chitosan microcapsule size was generally large, as can be seen in Table 2 under microcapsule size. This is because the concentration of chitosan solutions studied was high, as explained by the statement above. Specifically, an increase in concentration reduced the size of the microcapsules, which is consequent to higher conductivity. Flow rate is a significant independent variable on microcapsule size as shown in Table 3; its independent effect on microcapsule size alternated with chitosan solution conductivity, hence at low conductivity (concentration) at increasing flow rate, capsule size enlarged, while at high conductivity, capsule size reduced, which agrees with an observation in the literature [15]. An increase in voltage reduces microcapsule size [15,40], as also evidenced in this work.

The interaction effect of concentration/flow rate on the microcapsule size may be considered slight, as shown in Figure 2a. Microcapsule size increased at lower concentrations and low flow rates then reduced as the concentration increased. In contrast, at the rising flow rate, size reduced and then increased with a further rise in flow rate. Simultaneously, at rising concentration, microcapsule size decreased at an increasing flow rate. Figure 2b shows the effect of concentration and voltage on microcapsule size. The particle size slightly shrunk, but largely reduced at increasing voltage, even at a rising concentration. Hence, particle size was significantly decreased at applied voltages. The effect of flow rate and voltage on the microcapsule size is illustrated in Figure 2c. At increasing flow rates, the particle size was decreased until at a specific flow rate and then increased again, while voltage continuously reduced capsule size as it rose. Both parameters simultaneously led to a reduction in particle size. The combined influence of concentration and distance is shown in Figure 2d and may be considered negligible. The same is the effect of flow rate and distance shown in Figure 2e, where flow rate had the major influence on capsule size. As depicted in Figure 2f, the combined impact of voltage and distance decreased capsule size, which was dominated by the rising voltage.

#### 3.3.2. Sphericity

**Figure 3:**
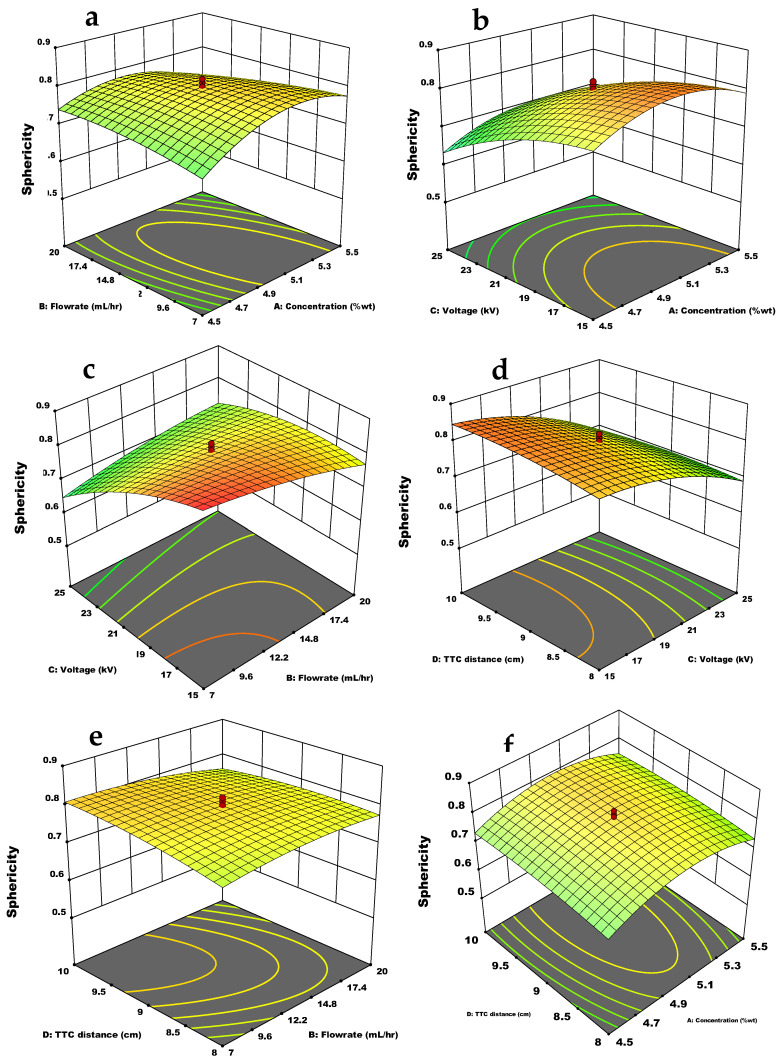
Response surface plot of the interaction effect of independent variables on sphericity: (**a**) Flow rate and concentration; (**b**) Voltage and concentration, (**c**) Voltage and flow rate; (**d**) TTC distance and voltage (**e**) TTC distance and flow rate; (**f**) TTC distance and concentration.

The effects of the studied parameters on sphericity are shown in Figure 3. Polymer concentration and flow rate are considered as main parameters that control the shape of the microcapsules [27]. As shown in Figure 3a, sphericity was affected by the simultaneous effect of flow rate and concentration; at low concentration and flow rate, sphericity decreased but increased at moderate effects of these parameters and then declined at their higher effects. At high concentrations and low voltage, the capsule appeared moderately spherical, as shown in Figure 3b, but drastically deviated from spherical as voltage grew. The best sphericity was observed at the lowest flow rate and voltage, as depicted in Figure 3c. Individually, distance offered no significant effect on sphericity in this study (Table 3). Figure 3d shows the combined effect of TTC distance and voltage on sphericity; at low voltage and longer distance, sphericity was better, but at a short distance and higher voltage, sphericity was reduced. The interaction between flow rate and distance is depicted in Figure 3e, which showed a slightly significant influence on sphericity. The combined influence of concentration and distance is shown in Figure 3f, where their moderate effects gave good sphericity.

#### 3.3.3. Yield

**Figure 4:**
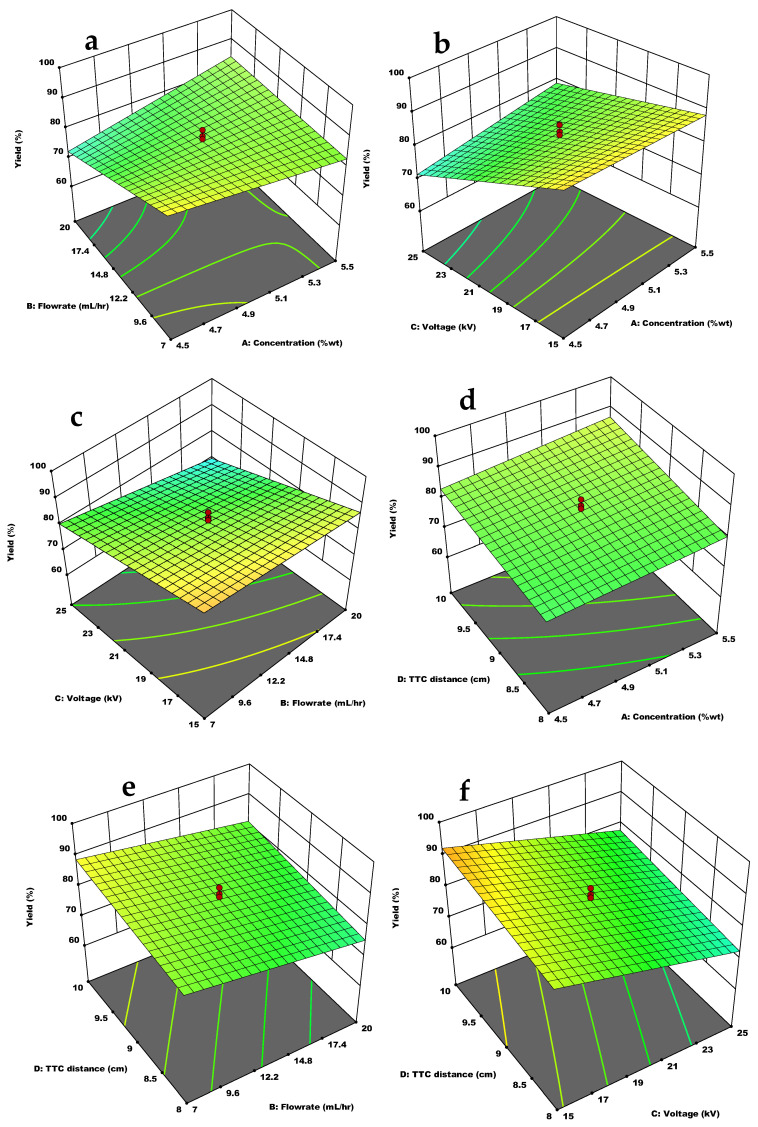
Response surface plot of the interaction effect of independent variables on yield: (**a**) Flow rate and concentration; (**b**) Voltage and concentration, (**c**) Voltage and flow rate; (**d**) TTC distance and voltage; (**e**)TTC distance and concentration; (**f**) TTC distance and flow rate.

Figure 4 shows the interaction effects of the independent variables on the yield of chitosan microcapsules. According to Figure 4a, at rising flow rates and concentration, the yield declined, but the highest yield was obtained at the lowest concentration and flow rates. The yield was adversely affected at high voltage and slightly increasing concentration, as shown in Figure 4b, leading to low microcapsule yield. From the interaction effects of voltage and flow rate in Figure 4c), high flow rates combined with high voltage produced declining yield. A good yield can be observed at low flow rates and voltages. The influence of concentration and TTC distance on yield was generally good at higher concentrations and longer distances, as depicted by Figure 4d. As TTC distance decreased at higher concentrations, yield reduced. Figure 4e shows that longer distances at lower flow rates favored yield, and short TTC distances at high flow rates negatively affected the yield of microcapsules. Low voltage and longer distance gave a significant yield, as shown in Figure 4f.

#### 3.3.4. Overall Effect of Coded Terms on the Responses Based on F-Value

**Figure 5:**
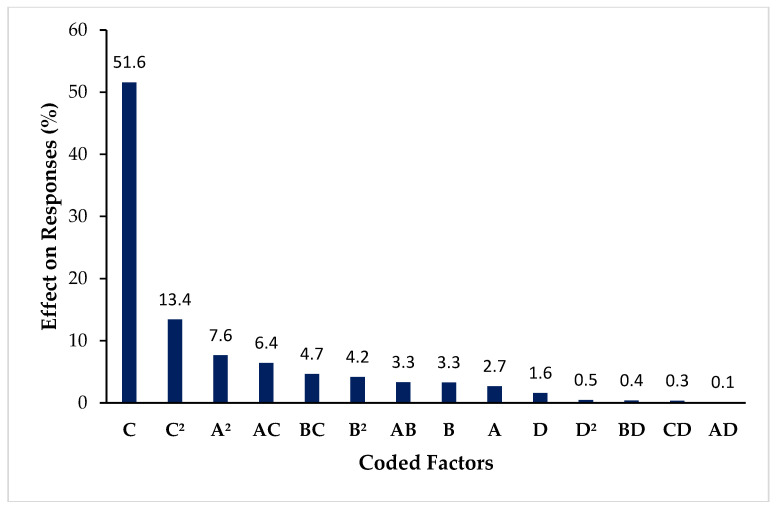
Pareto showing the effect of each coded term on all the responses (microcapsule size, sphericity, and yield).

Higher F-values greater than 4.0 confirms the significance of information or data. In this study, the overall influence, based on the F-value, of each coded term on the various responses is presented by the Pareto chart in Figure 5. The chart shows that voltage is ranked first with a significant effect on the responses compared to the other independent variable. Secondly, the flow rate with a percentage difference of 94%. The TTC distance showed the slightest effect. Considering the interaction terms, concentration and voltage affected the responses most, with an overall F-value of 128.8. In contrast, the interaction effect of concentration and TTC distance had no significant impact on the responses, with an overall F-value of 1.76, which is less than 4.0, the minimum significant F-value. This information helped in setting the goals for the optimization process.

### 3.4. Validation of the Model and Optimization of the Process

**Figure 6:**
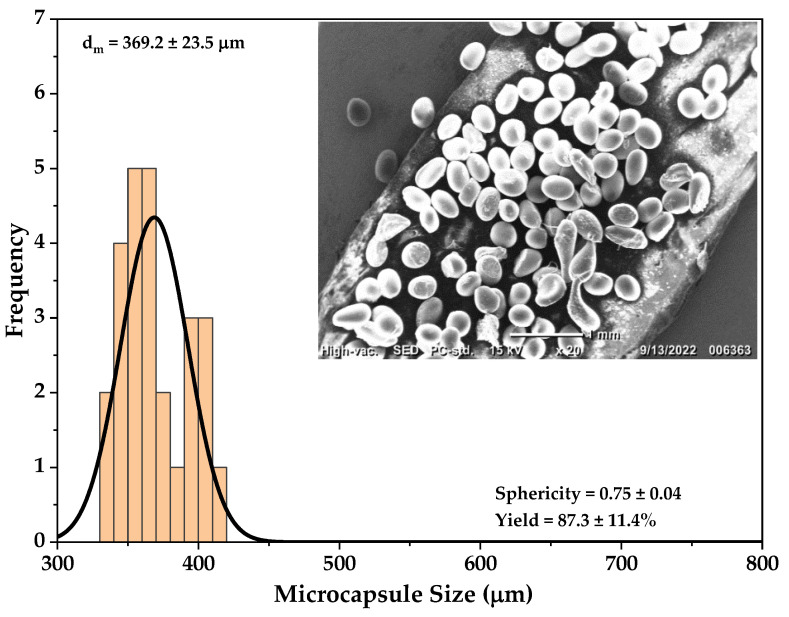
The microcapsule size distribution; the intersect is the SEM image of the obtained microcapsules at the optimized conditions.

Before optimization, the mean summary of the responses at the end of the experiments were 513 μm, 0.71, and 82% for microcapsule size, sphericity, and yield, respectively. Under the Design Expert numerical optimization, the independent variables’ goals were to minimize flow rate, maximize voltage, and minimize distance. Accordingly, the concentration was set in the range of 4.5 to 5.5 wt%. The goal set for the responses was to minimize, maximize, and maximize microcapsule size, sphericity, and yield, respectively. The desirability function in Design Expert, which has values between 0 and 1, provides the best set of operating parameters that can specifically generate the required performance level of responses. A desirability function of 0 represents undesirable and 1 is desirable. This function was used to compute the optimum values for the studied parameters based on the predetermined goals. Employing RSM, the optimum parameters for microcapsule size, sphericity and yield were selected at an 80% maximum desirability as the most desirable for the optimum parameters, that is, solution concentration = 5 wt%, flow rate = 7 mL/h, voltage = 22 kV and TTC distance = 8 cm. Table 7 shows the predicted and experimental results after carrying out the electrospraying experimental runs, in triplicate, based on the optimized conditions suggested by the software. In addition, the capsules size distribution and visual sphericity at the optimized conditions is given in Figure 6.

### 3.5. Comparing Present Chitosan Microcapsules with Others Produced and Cross-Linked via Electrospraying Technique

As mentioned earlier, chitosan microcapsules fabricated using the electrospraying technique are usually collected with a cross-linking solution or a flat surface of steel plate or aluminum foil. The properties of chitosan microcapsules produced in this study compared to those previously reported are presented in Table 8. Comparison is limited to capsules fabricated via the electrospraying method and collected with a cross-linking solution. The report on the yield of chitosan microcapsules via this method is highly sparse, and no previous report exists on sphericity to the best of the authors’ knowledge. However, there are a few reports on microcapsule size for chitosan microcapsules cross-linked with TPP, which is the only cross-linker reported so far for electrospraying of chitosan microcapsules.

## 4. Discussion

Together, predicted R^2^ and Adjusted R^2^ show how well the model matched the empirical results. In this study, the analysis of variance (ANOVA) results (Table 3 and Table 4) showed that it well represented the experimental data with quadratic models for microcapsule size and sphericity, and 2FI model for yield, with adequate coefficients of determination (R^2^) for the responses. The proximity of R^2^ to unity, in this study, shows that the effect of the independent variables on the response variables could be well explained through a quadratic and 2FI polynomial model. In addition, the difference between predicted and adjusted R^2^ with a value less than 0.2 implies that the models fit the experimental data well. Through the ANOVA, significant levels for the coefficients of the polynomial models are found; smaller *p*-value and larger F-value showed a significant effect of any term on the response variables [33].

A second-order response surface formula was described in determining the influence of the studied factors on microcapsule size. The model was significant with a *p*-value of <0.0001 and an F-value of 84.46, meaning that experimental data is well represented with a quadratic polynomial model for microcapsule size with a coefficient of determination (R^2^) value of 0.9875. A lack of fit value of 0.2761 (>0.05) demonstrated that residuals resulted from random errors and were insignificant [48]. Individual effects of the independent variables on the microcapsule size are significant except for TTC distance, which showed a non-significant effect with a higher *p*-value and smaller F-value. Thus, the singular effect of TTC distance in the model is not significant in determining microcapsule size. Additionally, testing the quadratic polynomial model predicted for sphericity by utilizing the difference between the adjusted R^2^ (0.9394) and predicted R^2^ (0.8601) showed its validity. An estimated difference of less than 0.2 (0.079) between the adjusted and predicted R^2^ verified the model’s validity. The *p*-value shows that the model is highly significant, thus proving that the independent variables are considerable terms for the model. The ANOVA results also depicted that the singular influence of independent variables such as concentration, flow rate, and TTC distance on the predicted sphericity model is not pronounced compared to their interaction effects. In addition, the fitness of data to the model is significant, as shown by the lack-of-fit (0.4583). The independent variables were significant model terms for the 2FI model of the yield, indicated by the model’s *p*-value (<0.0001). All the singular terms had a significant impact on the predicted model for the yield compared with interactive terms, except for Y_1_Y_2_, Y_1_Y_3_, and Y_2_Y_3_ terms, which showed a lower level of significance. The statistical fitness of the model, as verified by the difference between predicted and adjusted R^2^, proved it valid.

Chitosan microcapsules, cross-linked with the newly developed cross-linker, were successfully prepared by electrospraying technique using the different levels of the independent variables according to the experimental design. The microcapsule size of the prepared chitosan microcapsules was highly dependent on voltage, concentration, and flow rate. This is clearly shown by the significant effect of these parameters on the microcapsule size as indicated by the *p*-values (<0.05) in the ANOVA analysis and regression model, excluding TTC distance, which had an insignificant effect (with *p*-value > 0.05). It was reported in the literature that an increase in the distance leads to the production of small-sized capsules because of solvent volatilization from a long travel time (low flow rate) [7]. An opposite observation for flow rate and TTC distance was made in another literature [30], where the flow rate was insignificant while TTC distance was relevant. In another literature [27], where distance was held constant, the flow rate was influential according to the ANOVA analysis. This variation was attributed to acetic acid (solvent) concentration, i.e., longer distance allows solvent evaporation and vice versa [30], which means that when solvent concentration is minute, the TTC distance (varied) may not influence the particle size as evidenced in this work. In addition, the distance was not widely varied, which may be the reason for its independent insignificant effect on microcapsule size in this work. The interactive terms that showed a significant effect on the microcapsule size are only concentration/voltage and flow rate/voltage. Increasing flow rate leads to the formation of large microcapsule size because, at a high jet rate, the polymer quantity drips from the needle is higher [15,34]. It was explained in the literature that the rise in chitosan concentration increases the solution viscosity, leading to an increase in capsule size. Moreover, the increment in the polymer concentration enhances the solution conductivity [15,30] and reduces the microcapsule size [15]. In this work, the microcapsule size decreased with increasing chitosan concentrations, which may be attributed to the relative solution conductivity improvement with chitosan solution concentration, given that chitosan is a polyelectrolyte. This behavior is in agreement with the observation recorded in the literature [15]. The relative improvement in the solution conductivity may also be due to the low acetic acid concentration used. High acetic acid concentration decreases conductivity, which was observed to be the only solution parameter influenced by acetic acid [49]. Generally, the chitosan concentrations studied range was high, and the viscosity variation is narrowly varied, so the influence of viscosity on capsule size is negligible. Moreover, since the chitosan solution is positively charged, the electric field strength may cause the jet droplets to possess excess charge leading to droplet fragmentation and microcapsules of smaller sizes [15,18]. Therefore, the more relatively solution conductivity enhancement via increasing polymer concentration, the more charge is carried by the jet to be electrosprayed because of electrostatic repulsions, which then causes the jet to possess excess charge leading to droplets breaking into smaller sizes [4,15,30]. At high voltage, the microcapsule size is reduced because the production of droplets is favored, and the cone-jet, known as the Taylor cone, is stable at a higher voltage. Besides, a further voltage increase may lead to jet cone instability, producing a high polydispersity of microcapsules [50]. In addition, the surface charge density of the electrosprayed droplets increases at rising voltage, causing the droplets to disintegrate into smaller sizes [15].

The interactive effect of concentration and flow rate decreased microcapsules size (Figure 2a). Simultaneously, at rising concentration, there was a decline in microcapsule size at an increasing flow rate. This inverse effect is due to the rise in chitosan solution conductivity, which intensifies as concentration increases [15]. As reported previously, the rise in flow rate enlarges microcapsule size because of the larger polymer quantity that drips from the needle when the jet rate is higher [49]. Still, the combined effect of concentration and flow rate reduced microcapsule size. For instance, at 4.5 wt% and 15 mL/h, the microcapsule size was 450 μm, and at 5.5 wt% with 19.4 mL/h, the microcapsule size was 396 μm. This effect may be because of the increased conductivity of the chitosan solution at increasing concentrations since chitosan is a polyelectrolyte, which, when combined with moderate flow rates, allows the breaking of the droplets into smaller sizes [15]. Although, it was reported that larger capsules could be formed if flow rate and conductivity grew gradually [50].

The effect of concentration and voltage on microcapsule size (Figure 2b) led to reduced microcapsule size. As concentration increased, the particle size slightly decreased but largely declined at increasing voltage, confirming the observation made in a literature [15]. Still, this statement negates the observation made in another literature that there was no significant effect on capsule size at increasing concentration because the strong intermolecular interactions between chitosan and TPP at increasing chitosan concentration impeded the rise in capsule size [18]. In addition, a higher concentration raises the particle size, as it raises the solution viscosity [24]. This means that for the present work, an increase in conductivity through increasing concentration was more effective for capsule size reduction, as reported elsewhere [4]. At increasing voltage, particle size was significantly decreased, even at increasing concentrations. For the high voltage parameter, an increase in voltage causes a decrease in particle size, as observed in previous results [15,18,34]. Thus, the combined action of these parameters (concentration and voltage) produced smaller microcapsules. The maximum microcapsule size was obtained at low concentration and voltage, while the highest concentration and voltage produced particle size as small as 200 µm. The combined effect of flow rate and voltage (Figure 2c) exerted a quadratic effect on the microcapsule size; at increasing flow rates, the particle size was reduced until at a specific flow rate and then increased again. This may be due to the conductivity of the chitosan solution, which allows the breaking of droplets [4,15], as mentioned earlier. As the flow rate increased, the conductivity effect must have been overcome. Thus, the increasing particle size agreed with previous reports [15,18,34]. However, the combined effect of both parameters led to a reduction in particle size, showing that the effect of voltage must have overpowered that of the flow rate. The interaction influence of concentration and distance on the microcapsule size was slight and may be considered negligible in this case. The same is the effect of flow rate and distance because flow rate was the main factor affecting microcapsule size and was more influential. The capsule size diminished and then increased with a further increase in the flow rate. The combined impact of voltage and distance was majorly influenced by voltage, and at a decreasing distance and increasing voltage, the size was reduced.

Among all the independent variables studied, the voltage had a prime individual influence on sphericity. Flow rate and concentration are parameters that enforce gravity and viscosity-related forces. When applied in extreme conditions, they can cause non-spherical shapes like oblong, tear, or pear shapes [28]. This study’s combined effect of these parameters on the sphericity of chitosan microcapsules was significant. It has been reported that at low concentrations, deformation occurs because beads collide with the gelling bath and get disrupted. At higher concentrations, minimum sphericity can be observed, which is amplified by higher flow rates [28]. The deviation from sphericity at low and high flow rates and concentrations was not too wide (Figure 3a). The sphericity was around 0.7 and 0.74, respectively, at the abovementioned conditions, while the highest sphericity for chitosan microcapsule was observed in between. This may be because the studied concentrations are not wide apart and were sufficient to establish the required entanglement for microcapsule formation. The singular effect of voltage was highly significant in this study. The lowest sphericity is observed at high voltage (Figure 3b,d,f). At high concentrations and low voltage, the capsule appeared moderately spherical, but as the voltage increased, the capsule drastically deviated from spherical to non-spherical shape (Figure 3b). The best sphericity was observed at the lowest flow rate and voltage (Figure 3c). However, it was reported that a low flow rate had a negligible effect on sphericity [31]. Hence voltage dominated the effect on sphericity of microcapsule in its combined effect with flow rate in this study. Distance does not influence the sphericity of microcapsules, as earlier reported [5], and its singular effect on chitosan microcapsules is not significant in this work. Hence its effect on sphericity was slight and considered negligible because the increase in sphericity at an increasing distance was very small. It was reported that longer distances heightened the relaxation time of alginate droplets, thereby improving their sphericity [27]. This is true with the observation made in this work, even though the improvement was slight due to the closeness of the TTC distance varied to each other. In the interaction effect of TTC distance with voltage, voltage dominated the influence on sphericity (Figure 3d). The combined influence of concentration and distance on sphericity was not considered significant (Figure 3e) because the singular effect of TTC distance on sphericity is considered negligible, and the studied chitosan concentration was more dominant. As observed with the interaction between flow rate and distance (Figure 3f), there was a significant slight influence of these parameters on sphericity; sphericity slightly increased from low to moderate flow rates and TTC distance but decreased slightly with further increases. This decrease may come from higher concentration considered to dominate in the influence upon sphericity. On a general note, voltage offered the highest impact on the sphericity of chitosan microcapsule, as mentioned earlier.

Denser structures of the polymer and cross-linker encourage better yield by preventing the loss of capsules during the formation and collection processes [18]. Factors that can negatively affect the yield of microcapsules include loss of polymer solution through the needle, arc discharges and sputtering during electrospraying, and loss during washing of the formed capsules. The method employed in studying the effect of the independent variables is closer to that reported in a literature [18], where the collector is a cross-linking solution of TPP. In the case of this study, the collector is a newly formulated cross-linking solution, and the method was slightly modified, as already presented in Equation (5). All the studied parameters influenced the yield of chitosan microcapsules as presented in the ANOVA analysis (Table 4), and the effects of the interaction variables on yield are presented in Figure 4.

With growing flow rates, the yield decreased, which agrees with that reported earlier [15]. This may be because of sputtering of the solution resulting from a high flow rate or an increase in current due to a high flow rate, which leads to the detachment of fragmented droplets before reaching a stable jet mode. In addition, at rising concentrations, the yield declined. This may occur because, at high viscosity, there is resistance to the deformation and separation of the solution jet into droplets. The droplets are not allowed to break up, leading to a low yield. The highest yield was observed at the lowest concentration and flow rates (Figure 4a). In another work where lower concentrations were studied, the yield was improved at increasing concentration because the denser structure of chitosan reduced the loss at both formation and collection of capsules [18]. The yield reduced terribly at high voltage and a slightly increasing concentration (Figure 4b). Generally, the studied chitosan concentrations had a denser structure. Hence there was slight resistance to dripping at increasing viscosity leading to reduced yield at the introduction of higher voltage. Yield further decreased because of the formation of elongated capsules and fibers resulting from multiple unstable jets (arc discharge) [15]. From the interaction effects at low voltage and flow rate, the yield was high (Figure 4c). High flow rates combined with high voltage reduced the yield. This is attributed to other spraying modes, such as oscillating jet or multi-jet mode spraying, which may have developed, other than the cone-jet mode, given the high flow rate at high voltage. Additionally, a rise in the ejection speed may have limited the number of particles that find their way into the collector, as observed in a report [15]. Therefore, a good yield was observed at low flow rates and voltages. TTC distance played a significant role in the yield of capsules (Table 4). In the combined effect of concentration and TTC distance, the yield was generally good at higher concentrations and longer distances. This means that when other factors are held constant at higher concentrations and longer distances that allow complete solvent evaporation, there is proper contraction and solidification of electrosprayed capsules, thus, better yield. Adversely, microcapsules can be lost at longer distances when chitosan concentration is low due to the reduced intensity of the electric field power [4]. A longer distance at lower flow rates favored yield (Figure 4e). A longer distance allows for solvent evaporation and droplets fragmentation before reaching the collector, while low flow rates prevent erratic jet mode; hence yield is maximized. Low voltage and longer distance gave a significant yield (Figure 4f). This is contrary to the observation made by one study [15] where yield was better at a higher voltage. For this study, arc discharges were observed at higher voltage and shorter distances in the electrospraying of chitosan solutions, which led to reduced yield. This may be due to increased electric field influenced by high solution viscosity. Generally, the loss of capsules during formation and washing affected the yield of chitosan microcapsules in this study.

To confirm the model for validation, the suggested runs by the Design Expert were carried out in triplicate, and the obtained results were compared with the values predicted by the model with acceptable error margin (Table 7). At the predicted optimal conditions, the predicted size, sphericity, and yield were 386 µm, 0.72, and 80.6%. The capsule’s size distribution and visual sphericity were obtained at the optimized conditions (Figure 6). The experimental value for the size, 369.2 ± 23.5 µm, was lower than the predicted, 386 µm, but within the predicted deviation. Sphericity was also within range, 0.72 for the predicted and 0.75 ± 0.04 for the experimental. However, the experimental yield was 6.5% higher than the predicted one. This yield difference could be neglected since the suggested parameter was confirmed to be optimized, given that the experimental values were well within the boundaries of the predicted ones. Hence, it could be considered that the developed central composite design optimized the process successfully.

Comparing chitosan microcapsules produced in the present report to those reported in earlier works (Table 8) showed that capsule size ranged from 2.9 to 1100 μm depending on the chitosan solution concentration and other electrospraying conditions. For chitosan solution at 5 wt% cross-linked with TPP, the capsule size of 850 μm was obtained, while that obtained in the present report is 369 ± 15 μm at the same concentration. The highest yield reported for chitosan electrospraying was 83.1 ± 1.5%, while that obtained presently is 87.3 ± 8.4%, indicating improvement. Accordingly, the new presented cross linker is promising for the preparation of chitosan microcapsules.

## 5. Conclusions

The chitosan microcapsules were fabricated successfully using the electrospraying process and a novel collecting solution. The electrospraying process parameters such as voltage, polymer concentration, flow rate, tip-to-collector (TTC) distance, and their combined impacts were optimized using Design Expert. The cross-linking process was conducted using the collector solution. The collecting solution was a mixture of oxalic acid and sodium phosphate dibasic, which was used for the first time in this study in chitosan electrospraying. The process parameters were optimized statistically to obtain the impact of each parameter and their combined impacts. The optimization was evaluated according to the microcapsules’ size, sphericity, and yield. The voltage parameter showed the most significant impact among the studied parameters. On the other hand, the TTC distance showed a slight effect on the different responses, which means it could be neglected to be studied as a parameter. The conducted statistical optimization was evaluated by the experimental investigation of the predicted responses at the desired parameters. According to capsule size and sphericity results, a significant agreement was noticed between the predicted and the experimental results. The optimization model helped manufacture 369.2 ± 23.5 µm chitosan microcapsules instead of the experimentally designed 513 µm. The produced chitosan microcapsules had 369.2 ± 23.5 µm capsule size, 0.75 ± 0.04 sphericity, and 87.3 ± 11.4% yield at the optimized experimental conditions. The RSM-utilized optimization process was successfully examined for chitosan microcapsules manufacturing.

## Figures and Tables

**Table 1:** Experimental design factors and coded levels for chitosan microcapsules.

Independent Variables	Symbol	Coded Levels				
		−α	−1	0	+1	+α
Concentration (wt%)	A (Y_1_)	4.00	4.50	5.00	5.50	6.00
Flow rate (mL/h)	B (Y_2_)	0.50	7.00	13.50	20.00	26.50
Voltage (kV)	C (Y_3_)	10.00	15.00	20.00	25.00	30.00
TTC distance (cm)	D (Y_4_)	7.00	8.00	9.00	10.00	11.00

**Table 2:** Experimental design for chitosan microcapsules with independent variables and experimental values of responses.

Run	Independent Variables				Response Variables		
	Concentration (wt%)	Flow Rate (mL/h)	Voltage (kV)	TTC Distance (cm)	Microcapsule Size (μm)	Sphericity	Yield (%)
1	5	13.5	20	9	408.8 ± 18.4	0.79 ± 0.05	84 ± 10.0
2	4.5	20	15	8	764.1 ± 24.5	0.72 ± 0.04	79 ± 9.5
3	5	13.5	10	9	1094.4 ± 20.4	0.82 ± 0.04	96 ± 7.1
4	5.5	20	15	8	491.1 ± 19.5	0.66 ± 0.01	92 ± 5.6
5	5	13.5	20	9	413.5 ± 26.2	0.80 ± 0.02	93 ± 7.5
6	4	13.5	20	9	513.0 ± 23.1	0.58 ± 0.04	76 ± 7.5
7	5	13.5	20	7	346.4 ± 18.4	0.76 ± 0.03	72 ± 12.6
8	4.5	20	25	8	388.4 ± 20.3	0.67 ± 0.05	57 ± 8.5
9	4.5	7	15	8	876.4 ± 15.8	0.74 ± 0.02	97 ± 7.9
10	5.5	20	25	8	456.1 ± 21.7	0.67 ± 0.03	82 ± 4.6
11	5.5	7	25	8	390.3 ± 23.9	0.59 ± 0.01	79 ± 5.1
12	5	13.5	20	11	350.5 ± 25.7	0.79 ± 0.04	97 ± 9.8
13	5	13.5	20	9	371.2 ± 15.4	0.81 ± 0.05	61 ± 5.7
14	4.5	20	25	10	338.3 ± 21.4	0.64 ± 0.06	63 ± 3.4
15	5	13.5	30	9	400.9 ± 25.2	0.57 ± 0.04	64 ± 6.4
16	6	13.5	20	9	405.4 ± 22.3	0.61 ± 0.03	82 ± 3.5
17	5.5	7	25	10	378.1 ± 18.4	0.62 ± 0.01	87 ± 5.2
18	4.5	7	25	10	337.3 ± 26.4	0.57 ± 0.03	87 ± 6.9
19	5	13.5	20	9	366.3 ± 25.2	0.82 ± 0.04	73 ± 9.2
20	5	26.5	20	9	525.2 ± 19.5	0.75 ± 0.01	82 ± 5.2
21	5.5	20	15	10	509 ± 16.5	0.69 ± 0.01	92 ± 8.0
22	5	13.5	20	9	407.7 ± 14.4	0.82 ± 0.03	78 ± 10.3
23	4.5	7	25	8	340.2 ± 26.0	0.51 ± 0.02	88 ± 4.5
24	5.5	7	15	8	581.1 ± 9.5	0.82 ± 0.04	80 ± 5.7
25	4.5	20	15	10	801.1 ± 17.6	0.73 ± 0.04	91 ± 7.5
26	5.5	7	15	10	700.6 ± 19.1	0.88 ± 0.05	92 ± 2.8
27	5	0.5	20	9	675.0 ± 23.8	0.78 ± 0.03	96 ± 6.4
28	5	13.5	20	9	428.0 ± 19.2	0.76 ± 0.04	86 ± 7.8
29	5.5	20	25	10	459.6 ± 23.9	0.61 ± 0.04	83 ± 8.6
30	4.5	7	15	10	886.4 ± 24.0	0.79 ± 0.04	96 ± 8.7

**Table 3:** Analysis of Variance (ANOVA) Results of Microcapsule size and Sphericity.

	Sum of Squares		df	Mean Square		F-Value		*p*-Value	
	MS ^a^	Sph. ^b^		MS	Sph.	MS	Sph.	MS	Sph.
Model	1.113 × 10^6^	0.2683	14	79,477.68	0.0192	84.46	33.13	<0.0001	<0.0001
Y_1_: Concentration	40,016.67	0.0022	1	40,016.67	0.0022	42.52	3.81	<0.0001	0.0698
Y_2_:Flow rate	14,113.50	0.0015	1	14,113.50	0.0015	15.00	2.60	0.0015	0.1276
Y_3_: Voltage	6.364 × 10^5^	0.1134	1	6.364 × 10^5^	0.1134	676.22	196.15	<0.0001	<0.0001
Y_4_: TTC distance	748.17	0.0018	1	748.17	0.0018	0.7950	3.18	0.3867	0.0949
Y_1_Y_2_	12.25	0.0116	1	12.25	0.0116	0.0130	19.98	0.9107	0.0004
Y_1_Y_3_	1.099 × 10^5^	0.0001	1	1.099 × 10^5^	0.0001	116.78	0.0973	<0.0001	0.7594
Y_1_Y_4_	1156.00	0.0001	1	1156.00	0.0001	1.23	0.0973	0.2852	0.7594
Y_2_Y_3_	28,561.00	0.0333	1	28,561.00	0.0333	30.35	57.59	<0.0001	<0.0001
Y_2_Y_4_	702.25	0.0039	1	702.25	0.0039	0.7462	6.75	0.4013	0.0201
Y_3_Y_4_	3782.25	0.0014	1	3782.25	0.0014	4.02	2.43	0.0634	0.1398
$Y_{1}^{2}$	6768.05	0.0846	1	6768.05	0.0846	7.19	146.20	0.0171	<0.0001
$Y_{2}^{2}$	71,225.19	0.0047	1	71,225.19	0.0047	75.69	8.04	<0.0001	0.0125
$Y_{3}^{2}$	2.116 × 10^5^	0.0256	1	2.116 × 10^5^	0.0256	224.86	44.18	<0.0001	<0.0001
$Y_{4}^{2}$	3895.05	0.0030	1	3895.05	0.0030	4.14	5.25	0.0600	0.0368
**Residual**	14,115.67	0.0087	15	941.04	0.0006				
Lack of Fit	10,996.33	0.0061	10	1099.63	0.0006	1.76	1.17	0.2761	0.4583
Pure Error	3119.33	0.0026	5	623.87	0.0005				
Cor Total	1.127 × 10^6^	0.2769	29						
**Std. Dev.**	30.68	0.0240							
**Mean**	513.40	0.7123							
**C.V. %**	5.98	3.38							

^a^ Microcapsule size. ^b^ Sphericity.

**Table 4:** Analysis of Variance (ANOVA) Results of Yield and Statistical Fitness Values.

	Yield								
	Sum of Squares	df	Mean Square	F-Value	*p*-Value				
Model	2217.87	10	221.79	30.99	<0.0001				
Y_1_: Concentration	51.04	1	51.04	7.13	0.0151	Statistical fitness			
Y_2_:Flow rate	345.04	1	345.04	48.21	<0.0001		MS ^a^	Sph.^b^	Yield
Y_3_: Voltage	1162.04	1	1162.04	162.35	<0.0001	R²	0.9875	0.9687	0.9422
Y_4_: TTC distance	198.37	1	198.37	27.72	<0.0001	Adj. R²	0.9758	0.9394	0.9118
Y_1_Y_2_	333.06	1	333.06	46.53	<0.0001	Pred. R²	0.9398	0.8601	0.8057
Y_1_Y_3_	85.56	1	85.56	11.95	0.0026	Adeq Precision	35.4094	20.6068	24.769
Y_1_Y_4_	3.06	1	3.06	0.4279	0.5209				
Y_2_Y_3_	39.06	1	39.06	5.46	0.0306				
Y_2_Y_4_	0.0625	1	0.0625	0.0087	0.9265				
Y_3_Y_4_	0.5625	1	0.5625	0.0786	0.7822				
**Residual**	135.99	19	7.16						
Lack of Fit	93.99	14	6.71	0.7992	0.6622				
Pure Error	42.00	5	8.40						
**Cor Total**	2353.87	29							
**Std. Dev.**	2.68								
**Mean**	82.27								
**C.V. %**	3.25								

^a^ Microcapsule size. ^b^ Sphericity.

**Table 5:** Mean Absolute Percentage Error (MAPE) Analysis of the Response Models.

MAPE Equation	Response Model MAPE Values		
	Microcapsule Size	Sphericity	Yield
$\frac{1}{Ν}\overset{Ν}{\underset{i=1}{\sum }}\left|\frac{Χ-Χ\prime }{Χ}\right|Χ\;100$	4.03%	2.07%	6.05%

**Table 6:** Regression coefficient values for chitosan microcapsules.

Regression Coefficient	Microcapsule Size	Sphericity	Yield
Intercept	399.33	0.8000	82.27
Y_1_: Concentration	−40.83 ***	0.0096	1.46 ***
Y_2_: Flow rate	−24.25 **	−0.0079	−3.79 ***
Y_3_: Voltage	−162.83 ***	−0.0688 ***	−6.96 ***
Y_4_: TTC distance	5.58	0.0087	2.87 ***
Y_1_Y_2_	0.8750	−0.0269 ***	4.56 ***
Y_1_Y_3_	82.88 ***	0.0019	2.31 **
Y_1_Y_4_	8.50	−0.0019	0.4375
Y_2_Y_3_	42.25 ***	0.0456 ***	−1.56 *
Y_2_Y_4_	−6.62 *	−0.0156	0.0625
Y_3_Y_4_	−15.37	−0.0094	−0.1875
Y^2^_1_	15.71 *	−0.0555 ***	
Y^2^_2_	50.96 ***	−0.0130 *	
Y^2^_3_	87.83 ***	−0.0305 ***	
Y^2^_4_	−11.92	−0.0105	

**Table 7:** The predicted and experimental response at the optimized conditions of the process.

Optimized Parameters				Responses Variables					
Y_1_: Chitosan Concentration	Y_2_: Flow Rate	Y_3_: Voltage	Y_4_: TTC Distance	Microcapsule Size (μm)		Sphericity		Yield (%)	
wt%	mL/h	kV	cm	Predicted	Experimental	Predicted	Experimental	Predicted	Experimental
5	7	22	8	386	369.2 ± 23.5	0.72	0.75 ± 0.04	80.6	87.3 ± 11.4

**Table 8:** Comparison between the properties of prepared chitosan microcapsules with literature cross-linked chitosan.

Polymer Concentration (wt%)	Microcapsule Size (μm)	Yield %	Cross-Linker	Reference
2	9.78 ± 0.45	83.5 ± 1.5	TPP	[18]
5	850	−	TPP	[25]
1	2.9 ± 1.7	61.7 ± 0.1	TPP	[41]
3	350	−	TPP	[34]
0.5	568.04 ± 81.68	−	TPP	[42]
3	350 ± 50	−	TPP	[43]
2	400	−	TPP	[44]
1	7.89 ± 0.67	−	TPP	[45]
1–3	200–1100	−	TPP	[46]
2	85 ± 10	−	TPP	[47]
5	369 ± 23.5	87 ± 11.4	New cross-linker	Present work

## Data Availability

All data are provided.
